# Genetically encoded BRET-activated photodynamic therapy for the treatment of deep-seated tumors

**DOI:** 10.1038/s41377-022-00729-4

**Published:** 2022-02-21

**Authors:** Elena I. Shramova, Stepan P. Chumakov, Victoria O. Shipunova, Anastasiya V. Ryabova, Georgij B. Telegin, Andrei V. Kabashin, Sergey M. Deyev, Galina M. Proshkina

**Affiliations:** 1 Shemyakin–Ovchinnikov Institute of Bioorganic Chemistry, Russian Academy of Sciences, 16/10 Miklukho-Maklaya Street, Moscow, 117997 Russia; 2 MEPhI (Moscow Engineering Physics Institute), Institute of Engineering Physics for Biomedicine (PhysBio), 31 Kashirskoe shosse, Moscow, 115409 Russia; 3 Prokhorov General Physics Institute, Russian Academy of Sciences, Vavilova, 38, Moscow, 119991 Russia; 4 Branch of Shemyakin and Ovchinnikov Institute of Bioorganic Chemistry, Russian Academy of Sciences, Prospect Nauki 6, Pushchino, 142290 Russia; 5 Aix Marseille University, CNRS, LP3, 163 Ave. De Luminy, Case 917, 13288 Marseille, France

**Keywords:** Biophotonics, Lasers, LEDs and light sources

## Abstract

Photodynamic therapy (PDT) is one of the most appealing photonic modalities for cancer treatment based on anticancer activity of light-induced photosensitizer-mediated reactive oxygen species (ROS), but a limited depth of light penetration into tissues does not make possible the treatment of deep-seated neoplasms and thus complicates its widespread clinical adoption. Here, we introduce the concept of genetically encoded bioluminescence resonance energy transfer (BRET)-activated PDT, which combines an internal light source and a photosensitizer (PS) in a single-genetic construct, which can be delivered to tumors seated at virtually unlimited depth and then triggered by the injection of a substrate to initiate their treatment. To illustrate the concept, we engineered genetic NanoLuc-miniSOG BRET pair, combining NanoLuc luciferase flashlight and phototoxic flavoprotein miniSOG, which generates ROS under luciferase-substrate injection. We prove the concept feasibility in mice bearing NanoLuc-miniSOG expressing tumor, followed by its elimination under the luciferase-substrate administration. Then, we demonstrate a targeted delivery of NanoLuc-miniSOG gene, via tumor-specific lentiviral particles, into a tumor, followed by its successful elimination, with tumor-growth inhibition (TGI) coefficient exceeding 67%, which confirms a great therapeutic potential of the proposed concept. In conclusion, this study provides proof-of-concept for deep-tissue “photodynamic” therapy without external light source that can be considered as an alternative for traditional PDT.

## Graphical abstract

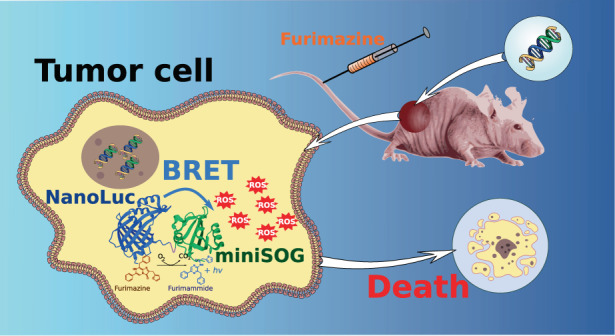

## Introduction

Photodynamic therapy (PDT) presents one of exciting photon therapy implementations, which can combine high efficiency and point localization of therapeutic action^1–3^. In PDT, the therapeutic effect is achieved via the employment of photosensitizers (PS) of organic (porphyrins, phthalocyanines, etc.) or inorganic (e.g., TiO_2_, CdSe/CdS, ZnO nanoparticles) origin, which are capable of generating reactive oxygen species (ROS), including singlet oxygen and free radicals, under photoexcitation, causing the damage of DNA, proteins and lipids of cancer cells^2–4^. PS are typically targeted to the tumor and then illuminated by light to initiate its elimination, while a high efficiency of PDT is due to a short lifetime in live cells (~3 µs for singlet oxygen in aqueous environment^5–8^) and short range of action of ROS, which leads to a selective cancer cell kill minimizing the damage of normal tissues. In addition, since the mechanism of action is different from those of alternative approaches, including chemotherapy and immunotherapy, PDT can be efficiently used in combination therapies to enhance the therapeutic outcome. However, due to a limited penetration of light into tissues^4,9^, PDT had a limited success so far and has been clinically adopted only for the treatment of dermatological diseases and epithelial tumors^10^. To overcome this “Achilles heel” of PDT, a series of approaches based on the use of near-infrared radiation-excited PS, up-conversion nanoparticles, X-rays and γ-rays have been proposed^9,11,12^, but these methods are still not free of major challenges and limitations. Indeed, the penetration depth of infrared light is still not very large (typically, less than 5 mm), while potential problems of up-conversion nanoparticles, infrared, X-ray and γ-ray sensitizers of ROS include systemic toxicity, hydrophobicity and fast aggregation.

The employment of internal light sources based on chemoluminescence or bioluminescence of some molecules in the presence of appropriate substrate or catalyst presents a radical solution of the limited light penetration problem^9,12^. Presenting a non-radiative energy transfer from a donor luciferase-substrate reaction to the acceptor-fluorophore, Bioluminescence Resonance Energy Transfer (BRET) looks especially promising to internally activate PS and thus implement deep self-illuminating PDT. Carpenter et al.^13^ showed the that excitation of PS hypericin mediated by luciferin results in time-extended intense-emission of PS, which is enough to induce virucidal activity. Later studies reported the development of a series of BRET-based agents for PDT, including *Renilla reniformis* luciferase conjugated to polymer-coated CdSe/ZnS quantum dots (QDs) emitting at 655 nm (QD655-RLuc8)^14,15^, Renilla luciferase conjugated to Rose Bengal^16^, Renilla luciferase (Rluc8) conjugated carboxylated QD655 and chlorin e6^17^, ferritin-luciferase nanoplatform conjugated to zinc (II)-protoporphyrin IX^18^, firefly luciferase conjugated with Rose Bengal in complex with biodegradable poly(lactic-co-glycolic acid) (PLGA) nanoparticles^19^, etc. Such compound agents promise the treatment of deep-seated tumors, but their design and applications still face major challenges. First, presenting complex chemical compounds combining the source and PS of different origin (typically, organic and inorganic), such constructs require thorough optimization and stabilization, as any misalignment of the gap between the constituents and their mutual orientation can radically drop their efficiency. In addition, the synthesis of these agents requires complicated and costly protocols, while most used compounds included non-biocompatible substances such as highly toxic Cd-containing QDs, which strongly complicates clinical applications prospects.

Here, we introduce the concept of fully genetically encoded PDT, which does not follow the current paradigm of BRET-activated internal PDT implying a chemical assembling of the source-PS conjugate. Instead, it combines the light source and PS in a single-gene-encoded construct, which can be delivered to a deep-seated tumor and triggered by the addition of an appropriate substrate to initiate PDT. The concept is illustrated using a home-designed NanoLuc-miniSOG platform, combining NanoLuc luciferase as the flashlight and phototoxic miniSOG as the PS, which is triggered by the injection of luciferase-substrate furimazine. First, we demonstrate the feasibility of genetically encoded PDT by expressing suicidal NanoLuc-miniSOG pair in BT-474 tumor cells implanted in mice, followed by the tumor elimination. To demonstrate the feasibility of targeted genetically encoded PDT, we then use pseudotyped lentiviral particles, exhibiting a high tropism to human epidermal growth receptor 2 (HER2), to deliver the NanoLuc-miniSOG gene into a HER2-positive tumor, followed by its elimination in the absence of external light. The proposed concept opens up avenues for the PDT-based treatment of deep-seated tumors, complimented by the possibility of image-guided therapy profiting from bioluminescence emission from NanoLuc.

## Results

### Description of NanoLuc-miniSOG complex for genetically encoded PDT

**Fig. 1:**
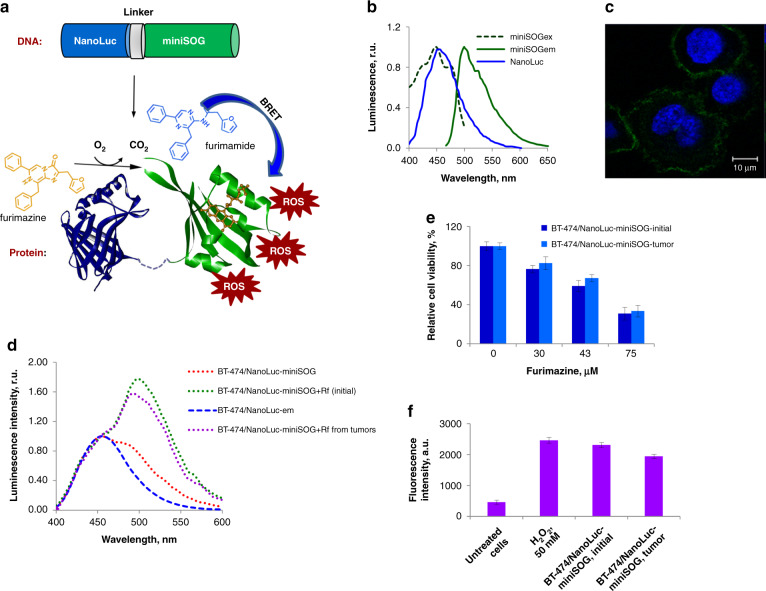
NanoLuc-miniSOG platform for genetically encoded BRET-activated PDT. **a** Schematic presentation of the concept. NanoLuc luciferase is fused to miniSOG phototoxic protein at genetic level. The energy generated by luciferase-substrate furimazine upon its oxidation by NanoLuc is nonradiatively transferred to miniSOG resulting in its excitation and generation of ROS, which in turn kill cancer cells. **b** Normalized emission spectrum of oxidized NanoLuc substrate (NanoLuc, blue line), normalized excitation (mSOG_ex_, green dotted line) and emission (mSOG_em_, green line) spectra of miniSOG. **c** Confocal microphotograph of BT-474-based stable cell line with NanoLuc-miniSOG located in the plasma membrane. Fluorescence signals in green (miniSOG) and blue (Hoechst 33342) channels are presented. **d** Normalized emission spectra of BT-474/NanoLuc-mem (blue dotted line) and BT-474/NanoLuc-miniSOG-mem initial cell lines (green dotted line) and BT-474/NanoLuc-miniSOG isolated from the tumor (magenta dotted line) in the presence of 75 μM furimazine and riboflavine and BT-474/NanoLuc-miniSOG in the absence of riboflavin (red dotted line). **e** In vitro cytotoxicity studies of NanoLuc-miniSOG activated by BRET. The data of MTT assay are presented for BT-474/NanoLuc-miniSOG initial cell line and BT-474/NanoLuc-miniSOG from the tumor in the presence of different furimazine concentration. The data are shown as the mean ± SD (*n* = 3). **f** ROS production in NanoLuc-miniSOG BRET-activated system. Fluorescence intensity of DCF, an indicator of ROS, in BT-474/NanoLuc-miniSOG initial cell line and BT-474/NanoLuc-miniSOG from the tumor in the presence of 75 μM furimazine are presented. The measurement performed at 529 nm. The data are shown as ±SD (*n* = 3)

To design an efficient BRET pair, one has to achieve a significant overlap between the bioluminescent emission spectrum generated by luciferase enzymes and the absorption spectrum of the acceptor, while the gap between donor and acceptor components should be minimal (typically, within 10–100 nm range). These conditions can be satisfied by the employment of genetically encoded NanoLuc-miniSOG protein complex, composed of small (19 kDa) ATP-independent luciferase NanoLuc derived from deep-sea shrimp *Oplophorus gracilirostris*^20^ as the energy donor, and a fluorescent phototoxic protein miniSOG^21,22^, which uses flavin mononucleotide (FMN) as a cofactor. The schematic presentation of NanoLuc-miniSOG complex for genetically encoded PDT is shown in Fig. 1a. The emission spectrum of oxidized NanoLuc substrate furimazine is around 460 nm (blue line on Fig. 1b), which substantially overlaps with the absorption spectrum of miniSOG (*λ*_max_ 448 nm, green dotted line on Fig. 1b). Therefore, if the two proteins are fused (for example, at the genetic level) the excited-state energy of the oxidized reaction intermediate in the NanoLuc active site could be transferred to excite miniSOG by BRET^23^. Riboflavin mononucleotide (Rf) was used as FMN source. When penetrated through the cellular membrane, Rf turns into FMN during phosphorylation by riboflavin kinase. Because of FMN cofactor, blue light is absorbed by miniSOG (with maximum absorption at 448 nm and a shoulder at 473 nm), while the fluorescence takes place in the green spectral region with the maximal emission at 500 nm and a shoulder at 528 nm. Also due to FMN, miniSOG can produce ROS via the type I and the type II photoreactions^21,22,24,25^ under exposure to blue light.

### Expression of NanoLuc-miniSOG pair in BT-474 tumor cells

In this study, NanoLuc-miniSOG protein complex was expressed in BT-474 cell line, which exhibit a relatively low sensitivity to the toxicity of luciferase-substrate furimazine upon a long-term exposure to the substrate^26^. Since BRET-activated NanoLuc-miniSOG cytotoxicity is dependent on intracellular localization^27^, BT-474-based stable cell line with NanoLuc-miniSOG located in plasma membrane was created, as described in Materials and methods section. The membrane localization of NanoLuc-miniSOG was confirmed with the confocal laser scanning microscopy. As we can see from Fig. 1c, under blue irradiation (448 nm) cells’ membranes have gentle green corona.

To evaluate fluorescence-bioluminescence properties of BT-474 cells expressing NanoLuc-miniSOG, we recorded the emission spectra (400–600 nm) of living cells under 75 μM of NanoLuc substrate furimazine. As one can see from Fig. 1d (green dotted line), a strong luminescence peak at 500 nm associated with photo-exited miniSOG appeared only in the case of cells incubated under 150 μM Rf. We were unable to detect emission at 500 nm if BT-474/NanoLuc-miniSOG were cultivated without Rf (red dotted line on Fig. 1d). This result strongly confirmed the occurrence of BRET. The BRET ratio can be calculated by the comparison of intensities of bioluminescence signals with/without Rf. Using a method described in Materials and Methods section, the BRET ratio was found to be equal to 0.74 ± 0.05, which is consistent with our previous data^23^.

In vitro photodynamic cytotoxicity was investigated by a cellular MTT assay. Our data confirmed that the incubation of BT-474/NanoLuc-miniSOG cells under luciferase-substrate and Rf led to obvious cytotoxicity correlated with furimazine concentration (Fig. 1e). Cytotoxic effect was equal to 71% at the presence of 75 μM furimazine.

To estimate intracellular ROS production during BRET-activation of miniSOG in BT-474 cell line, a ROS measurement in live cells expressing NanoLuc-miniSOG was performed. In photochemical reactions induced by miniSOG, the production of oxygen-dependent radicals (H_2_O_2_ and superoxide anion) prevails over singlet oxygen production^24,25^. That is why for intracellular ROS evaluation we used H_2_O_2_-sensitive dye 5-(and-6)-carboxy-2′,7′-dichlorodihydrofluorescein diacetate (carboxy-H_2_DCFDA). Owing to a non-ionic and non-polar nature, carboxy-H_2_DCFDA can penetrate through the lipid bilayer of the cell membrane and be converted by esterases into H_2_DCF. In the presence of ROS, H_2_DCF oxidizes and converts into fluorescent carboxy-2′,7′-dichlorofluorescein (carboxy-DCF), whose fluorescence can be measured spectrophotometrically^28^. Therefore, carboxy-H_2_DCFDA probe can be considered as intracellular marker of ROS. In our tests, the fluorescence signal from carboxy-DCF was measured in BT-474/NanoLuc-miniSOG under 75 μM of furimazine (Fig. 1f). BT-474 cells treated with 50 mM H_2_O_2_ were employed as a positive control, while BT-474/NanoLuc-miniSOG furimazine non-treated cells were used as the negative control. As shown in Fig. 1f, a strong fluorescence signal at 529 nm (the emission maximum of carboxy-DCF) was detected in BT-474 cells treated with 50 mM H_2_O_2_ or BT-474/NanoLuc-miniSOG treated with furimazine but there was no significant signal in the case of BT-474/NanoLuc-miniSOG without furimazine.

### Characteristics of a primary cell culture created from a mouse tumor

To verify whether BT-474/NanoLuc-miniSOG cells maintain all the properties in the xenograft model compared to initial cells, subcutaneous tumors formed by BT-474/NanoLuc-miniSOG cells were extracted from animals on the 25th day after inoculation and transferred into a primary cell culture. After several passages when the culture was completely depleted from mouse fibroblasts, the cells were subjected to spectral analysis, as well as BRET ratio definition, capability for ROS generation, and in vitro cytotoxicity determination. As we can see from Fig. 1(d, e, f) all properties of the original BT-474/NanoLuc-miniSOG cell line were maintained in the cells isolated from the tumors. According to spectral analysis (magenta dotted line on Fig. 1d), NanoLuc-miniSOG spectrum with furimazine and Rf did not change in comparison to the one of the initial cell line (green dotted line on Fig. 1d) and the BRET ratio was equal to that of the original cell line: 0.77 ± 0.06 and 0.74 ± 0.05, respectively. The cytotoxic effects, as well as the capability for ROS production, were comparable with those for the initial (parent) cell line (Fig. 1e, f). These results strongly suggest that engineered BT-474 cell line expressing NanoLuc-miniSOG maintains all properties in tumor xenograft.

### Assessment of efficacy of NanoLuc-miniSOG system expressed by BT-474 cells in vivo

**Fig. 2:**
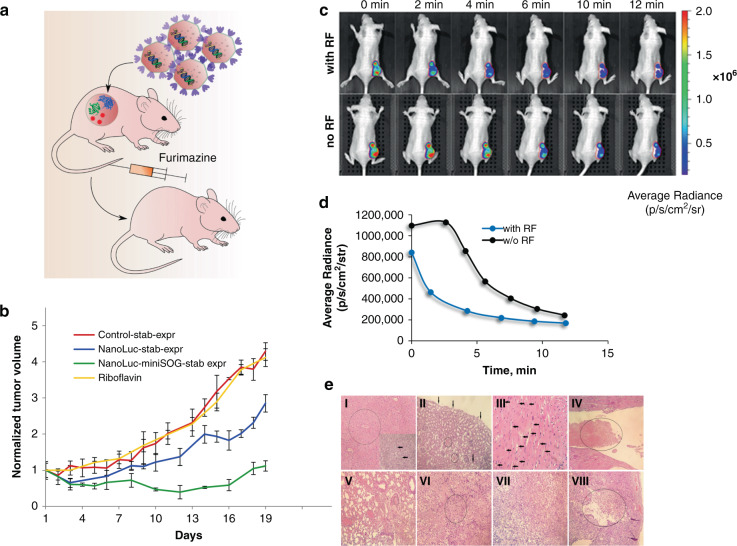
Assessment of efficacy of NanoLuc-miniSOG system expressed by BT-474 cells in vivo. **a** Schematic presentation of the experiment. BT-474 cells stably expressing NanoLuc-miniSOG are inoculated subcutaneously into an animal. Gene expression of NanoLuc-miniSOG leads to production of fusion protein NanoLuc-miniSOG, which leads to BRET-activation of miniSOG after the injection of furimazine, followed by the ROS production and cancer cell kill. **b** Tumor-growth curves for various treatment conditions: mice bearing BT-474 tumor xenograft, treated with PBS (red curve); BT-474/NanoLuc-expressing tumors treated with furimazine (blue curve); mice bearing NanoLuc-miniSOG-expressing tumors treated with furimazine and riboflavin (green curve); mice bearing NanoLuc-miniSOG-expressing tumors treated with riboflavin (yellow curve). Data are presented as the mean ± SD (*n* = 6). **c** Evidence of BRET effect in vivo. An image of a living animal imaging after i.v. furimazine administration with (top row) and without (bottom row) Rf pre-injection. The difference in the distribution of average luminescence signals is a sign of BRET in NanoLuc-miniSOG system. The tumor region is indicated as a red-circled area. **d** Luminescence intensity signal from the tumor region measured in photons per second per cm^2^ per steradian with and without RF pre-injection. **e** Hematoxylin-eosin-stained histological sections of different organs and tumors at the end of furimazine treatment (I-VI) and after completion of the experiment (VII, VIII). **I**—Liver. In the center of the lobules weakly expressed plethora and hydropic degeneration of hepatocytes are circled, necrosis of individual hepatocytes with small focal leukocyte infiltration are pointed with arrows, magnification ×200. **II**—Kidney with focal hydropic dystrophy of the epithelium of the proximal convoluted tubules (arrows) and small focal hemorrhages (circled), magnification ×200. **III**—Hydropic dystrophy of cardiomyocytes (arrows), magnification ×400. **IV**—Heart with a fibrin thrombus (circled) in the lumen of the left ventricle, magnification ×200. **V**—Lungs with plethora, confluent hemorrhages, fibrin thrombus in the lumen of the vessel (circled), magnification ×200. **VI**—Tumor with a focus of necrosis (circled), magnification ×200. **VII**—Tumor. Poorly differentiated adenocarcinoma, magnification ×200. **VIII**—Tumor with necrosis and decay cavity (decay cavity with necrotic masses in the lumen is circled), magnification ×100

To demonstrate BRET-activated PDT in vivo, engineered BT-474 cells stably expressing NanoLuc-miniSOG BRET-pair were used to generate tumors in BALB/c nu/nu mice, as shown schematically in Fig. 2a. Mice bearing BT-474 tumors or BT-474 tumors expressing NanoLuc gene were used as a negative control. After tumor volume reached ~100 mm^3^, the treatment was initiated according the followed scheme: BT-474-bearing mice were treated with 0.1 mL phosphate-buffered saline (PBS) daily; BT-474/NanoLuc or BT-474/NanoLuc-miniSOG-bearing mice were treated intravenously with 7 μg of furimazine and 10 mg/kg of Rf trice per day for 10 days. To estimate the impact of Rf on tumor growth, BT-474/NanoLuc-miniSOG-bearing mice were treated with Rf alone. Since miniSOG expressed in xenograft tumors cannot be used as a photosensitizer in combination with external blue light illumination, we did not run control test with miniSOG alone^29^.

We previously showed that daily intravenous administration of furimazine substrate in a single dose of 20 to 60 μg per animal for 7 days causes hydropic dystrophy and necrosis of hepatocytes, but the splitting of a dose of 20 μg into several injections (three times daily at 7 μg) reduces the hepatotoxicity of furimazine^26^. That is why for BRET-PDT experiment mice were injected with 7 μg furimazine thrice per day.

As presented in Fig. 2b, the BRET-PDT group demonstrated obvious tumor-growth inhibition with TGI 72% (green curve). Unexpectedly, the group of animals with the NanoLuc-bearing tumors revealed tumor-growth inhibition with TGI 30% (blue curve). RF itself at the concentrations used in the tests did not affect on tumor growth (yellow curve). On day 10, the BRET-mediated-PDT–treated group as well as NanoLuc-bearing group did not exhibit significantly reduced weight compared to the starting point of the experiment, suggesting the low general toxicity of furimazine. Based on this data we can state that NanoLuc-miniSOG BRET pair can be used in vivo for BRET-induced PDT.

### Evidence of BRET effect in vivo

To confirm the BRET in the developed NanoLuc-miniSOG system on animal model, a method of in vivo bioluminescence imaging using an IVIS Spectrum CT system was employed. A mouse with BT-474/NanoLuc-miniSOG xenograft tumor was injected with furimazine at the dose of 20 µg, and after 1 min bioluminescent signals were monitored with intervals of about 2 min (Fig. 2c, d). After 24 h the same mouse was pre-injected with a 10 mg/kg of Rf in 100 µL of PBS, and 1 h later, furimazine was injected at a dose of 20 μg, and the signals of bioluminescence were again recorded. When cells were saturated with Rf at the beginning of the detection, 1 min after the injection of furimazine, the detected signal gradually dropped and did not reach saturation, compared to the case of detection of the luciferase signal in the absence of Rf. In the time region (from the 3rd min of detection) when the signal intensity turned to be a monotonic function, the absolute values of the average luminescence were about three times lower than that in the absence of Rf (Fig. 2d). These differences in the behavior of the average luminescence signals indicate an efficient process of energy transfer from the donor (NanoLuc luciferase) to the acceptor (miniSOG) and are direct and obvious evidence of BRET in vivo. Indeed, in animals received Rf, energy of excitation stage of oxidized form of furimazine was partially transferred on FMN bound noncovalently to miniSOG, that led to low intensity of luminescence (blue curve on Fig. 2d). In animals without Rf, miniSOG is in inactive stage, because without saturation with its cofactor FMN, miniSOG cannot absorb blue light and emit green fluorescence. That is why in this case, luminescence curve is much higher (black curve on Fig. 2d).

**Fig. 3:**
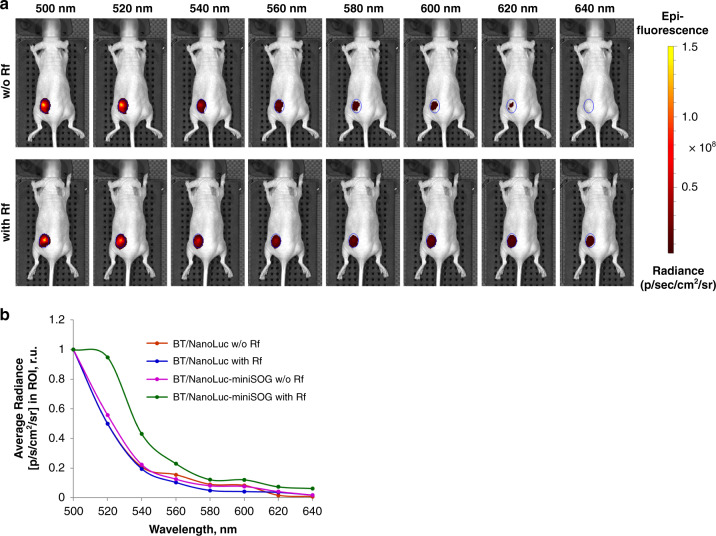
Evidence of NanoLuc-mediated BRET effect in vivo. **a** Fluorescence images of NanoLuc-miniSOG-tumor-bearing animals. Photos are performed after i.v. furimazine administration without (top row) and with (bottom row) Rf pre-injection. The ROI are indicated as a blue-circled area. **b** Normalized fluorescence intensity curves (in photons per second per cm^2^ per steradian) from the tumor region measured with and without RF pre-injection for mice bearing NanoLuc (blue and red curves, respectively) or NanoLuc-miniSOG-expressing tumors (green and magenta curves, respectively)

To prove NanoLuc-mediated BRET effect in vivo, we also used another approach based on a direct detection of fluorescence signal from exited miniSOG in NanoLuc-miniSOG tumor-bearing mice. In this experiment, mice with BT-474/NanoLuc-expressing tumors were used as a negative control. Using IVIS Specrum CT system, fluorescence signals were collected in the mode without excitation (Fig. 3a). As one can see from Fig. 3b, there was no emission peak between 500-520 nm (in the presence or absence of Rf) in the case of mice with tumors expressing NanoLuc. However, in mice bearing NanoLuc-miniSOG expressing tumors, a strong fluorescence peak corresponding to the miniSOG maximal emission appeared in animals injected with Rf. We believe that the result of this experiment gives strong evidence for NanoLuc-induced BRET effect in vivo.

### Histological studies of general toxicity

To assess general toxicity and the effectiveness of treatment, histological analysis of animals’ organs and tumors was carried out immediately after the termination of injections and at the end of the experiment (Fig. 2e). One day after the end of injections in the furimazine-treated group, mild signs of intoxication were noted in the form of focal hydropic degeneration of hepatocytes (Fig. 2e-I), nephrocytes (Fig. 2e-II), cardiomyocytes (Fig. 2e-II) and a moderate liver plethora (Fig. 2e-I) associated with the hepatotoxic effect and systemic toxicity of furimazine in vivo^26^. Necrosis of individual hepatocytes with small focal leukocyte infiltration (Fig. 2e-I) was also noted. Weak changes were observed in the lung parenchyma (Fig. 2e-V), where a mild venous plethora and single small focal hemorrhages were determined, which also evidences the nonspecific toxic effect of furimazine. Also, the presence of signs of DIC (Disseminated Intravascular Coagulation) in the form of fibrin blood clots in the vessels of the lungs (Fig. 2e-V) and heart (Fig. 2e-IV) was noted. It is also most likely a consequence of the toxic effect of furimazine. Visually definable damage in the spleen and lymph nodes was not observed. The tumors in all cases were poorly differentiated adenocarcinomas. Numerous foci of necrosis of various sizes (Fig. 2e-VI) were presented in the tumors immediately after the treatment stop. At the end of the experiment, the tumors of animals from experimental and control groups had foci of necrosis with the formation of a decay cavity, which is associated with a lack of nutrition of the tumor cells (Fig. 2eV-VII, VIII).

A pathomorphological study of all organs of animals from different groups at the end of the experiment did not reveal significant macroscopic and microscopic changes, with the exception of poorly pronounced signs of DIC, most likely due to the body’s response to the oncological state of the terminal stage. Thus, changes in various organs caused by the systemic toxicity of furimazine during treatment turned out to be reversible, with the exception of maintaining poorly pronounced signs of DIC.

### Targeted BRET-activated PDT based on pseudotyped lentiviruses bearing NanoLuc-miniSOG BRET pair

**Fig. 4:**
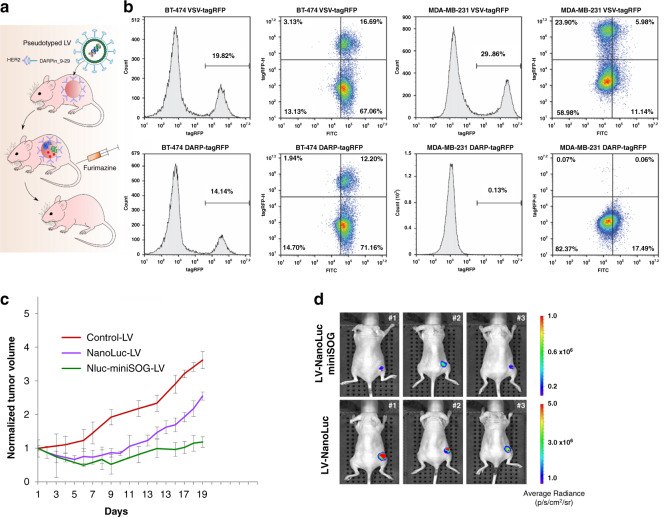
Targeted PDT using HER2-specific lentiviruses (LVs) as carriers of NanoLuc-miniSOG gene. **a** Schematic representation of experiment. HER2-positive tumor is targeted with HER2-specific LVs carrying the NanoLuc-miniSOG gene. DARPin_9-29 incorporated in LV’s envelope targets LVs to HER2-specific tumor. **b** Comparison of receptor specificity and transduction efficiencies of VSV-pseudotyped and HER2-retargeted lentiviral vectors. Histograms demonstrate similar transduction efficiencies of VSV-tagRFP LVs in HER2-positive and HER2-negative cell line, whereas DARPin_9-29-tagRFP LVs were able to exclusively transduce HER2-positive BT-474 cells. **c** Tumor-growth curves for control group (mice treated with PBS only), as well as for groups carrying NanoLuc or NanoLuc-miniSOG LVs particles and treated with furimazine. Data are presented as the mean ± SD (*n* = 6). **d** In vivo bioimaging of animals bearing HER2-positive tumors and injected i.t. with LVs-NanoLuc-miniSOG or LVs-NanoLuc. Three mice from each group on the 13th day after LVs injection are presented

Profiting from NanoLuc-miniSOG genetic nature, we used pseudotyped viral system to deliver created BRET-pair directly to a tumor. BT-474 cell line used in our work is characterized by overexpression of tumor-associated antigen HER2. HER2 is a receptor tyrosine kinase, which is strongly expressed by cancer cells of epithelial origin, such as pancreatic, breast, ovarian and colon, but its expression is low or absent in normal human tissues^30^. That is why this receptor is considered an excellent target in precision anti-HER2 therapy. We use lentiviral vectors pseudotyped with HER2-specific scaffold protein DARPin_9-29^31^ to deliver NanoLuc-miniSOG genetic construct into BT-474 tumor in the animal model (Fig. 4a). For this purpose, human immunodeficiency virus HIV-1 vector particles were produced in the presence of truncated measles virus (MV) glycoproteins (hemagglutinin HcΔ18 and fusion protein FcΔ30)^31^, moreover the C-terminus of MV hemagglutinin protein was modified with DARPin9_29 so that tropism for naturally recognized CD46 receptor is changed to HER2 receptor. DARPins (Designed ankyrin repeat proteins) are binding scaffolds of non-immunoglobuline nature that have been developed as an alternative to immunoglobulin-based proteins^32^.

To estimate in vitro the targeting potential of DARPin-LV particles, we transduced HER2-positive BT-474 and expressing HER2 at low level MDA-MB-231 cells with HcΔ18-DARPin9.29-pseudotyped reporter lentiviral vector carrying tagRFP expression cassette. LV particles pseudotyped with the glycoprotein (G) of the vesicular stomatitis virus (VSV), which mediates efficient transduction of most human cell types, were used as a control. Transductions were performed with multiplicity of infection (MOI) of 0.2, which yielded comparable transduction efficiencies for DARPin-LVs and VSV-LVs in HER2-positive BT-474 cells: 14.4% vs. 19.2% (Fig. 4b). VSV-LV transduced both types of cells with approximately equal efficiency (20–30%), whereas DARPin-LVs were able to exclusively transduce HER2-positive BT-474 cells (Fig. 4b). To prove HER2 expression on the cell surface, cells were stained with DARPin_9-29 conjugated with FITC (fluorescein isothiocyanate). As one can see from the histograms on Fig. 4b, FITC-positive population is 67–71% in BT-474 samples and 11–17.5% in MDA-MB-231 samples. Taken together, the results demonstrate high transduction efficiency and outstanding HER2 specificity of DARPin-LVs.

To evaluate in vivo the photodynamic potency of the HER2-specific LVs, carrying NanoLuc-miniSOG or NanoLuc genes, mice bearing BT-474 xenograft tumors were treated by intratumoral injections of viruses, when the tumors had reached 50 mm^3^. The treatment with furimazine and RF was started 3 days after the transduction.

As presented in Fig. 4c, the group of animals treated with HER2-specific LV-NanoLuc-miniSOG showed significant tumor-growth inhibition with TGI 67%. The group of animals treated with LV-NanoLuc particles revealed tumor-growth inhibition equal to 30% as in the case of BT-474/NanoLuc-tumor-bearing mice. Based on similar results obtained during these two different experiments, we propose that the oxidized product of furimazine (furimamide) can be toxic for tumor cells.

The efficiency of targeted BRET-mediated PDT in animals bearing BT-474 xenograft tumors and treated with HER2-specific LVs was proved by in vivo bioimaging. Figure 4d presents images of animals on the 13th day of treatment. One can see that the intensity of luminescence signal in animals treated with NanoLuc-miniSOG LVs (upper panel on Fig. 4d) is less than in the group treated with NanoLuc LVs (lower panel on Fig. 4d), which means that NanoLuc-miniSOG delivered by HER2-specific LVs efficiently kills cancer cells.

## Discussion

A weak penetration depth of light into tissues, even for pumping wavelengths matching the window of relative biotissue transparency (650–950 nm), is the main obstacle, which limits application prospects of classical PDT approaches in the treatment of deep-tissue tumors and their metastasis. The employment of bioengineered internal source-independent platforms based on BRET looks as the most promising pathway overcome this obstacle and advance PDT technology onto a new level. In this case, biocompatible light-emitting systems based on luciferase*substrate reactions could serve as a light source and an endogenous donor for BRET systems. However, currently available BRET complexes for PDT using chemically assembled compounds based on the conjugation of luciferase with quantum dots^14,15,17^ or other substances^16,18,19^ still have major problems for potential clinical applications, including the difficulty of structure optimization and stabilization, complicated and costly conjugation protocols, and finally toxicity of involved compounds (as an example, they often contain highly toxic Cd-based QDs).

By introducing the concept of genetically encoded BRET-activated PDT, we propose a viable solution of the bottleneck problem of limited treatment depth of PDT, while avoiding problems of currently available chemically assembled BRET complexes. For the demonstration of the concept, we designed a genetically encoded NanoLuc-miniSOG platform, which can be delivered by a properly selected carrier to a tumor, and then triggered by the injection of furimazine substrate. To assess the efficiency of this pair, we expressed NanoLuc-miniSOG BRET-pair in breast ductal carcinoma BT-474 cancer cells and then examined its operation in vitro and in vivo. Spectroscopic characterization of BT-474/NanoLuc-miniSOG cell line confirmed that BRET between NanoLuc and miniSOG indeed occurs with the BRET ratio reaching 0.74 ± 0.05 (Fig. 1d). The recorded ratio is in agreement with our previous data^23^, as well as with data for other BRET-based genetically encoded sensor systems^33,34^, confirming a high efficiency of the pair. We demonstrated by MTT assays that NanoLuc-miniSOG efficiently kills the cancer cells in the presence of furimazine (Fig. 1e), while other tests confirmed that the endogenous bioluminescence can serve as a light source to activate the miniSOG for ROS generation (Fig. 1f). Using the model of mice bearing engineered BT-474 cells expressing NanoLuc-miniSOG, we recorded TGI exceeded 72% after the injection of furimazine without external light irradiation (Fig. 2). It means that the inhibition of tumor growth occurs due to BRET-induced PDT, although cytotoxicity of oxidized product of furimazine could also partially contribute to the cell death (Fig. 2b). It is also important that bioluminescence imaging can be used in our case to assess BRET efficiency in vivo. The differences of the average luminescence signal with or without Rf in animals evidenced an efficient process of energy transfer from the donor (oxidized NanoLuc luciferase-substrate) to the acceptor (miniSOG) in the case of tumor Rf saturation (Fig. 2c, d). Finally, genetic nature of proposed NanoLuc-miniSOG BRET-pair allowed us to use targeted viral system for gene delivery precisely into tumor (Fig. 4). Using HER2-specific LVs carrying NanoLuc-miniSOG gene we achieved significant regression (TGI 67%) of HER2-positive xerograph tumor in the animal model, confirming a high efficiency of the proposed concept.

We foresee several major advantages of the proposed concept of genetically encoded BRET-activated PDT over currently present chemically assembled BRET-based systems for PDT. First, the genetic nature of functional PDT elements provides an ideal spatial architecture of the BRET construct, conditioning optimal distance between the internal triggering source and the PS, which is obviously very difficult or impossible in the case of chemical assembling. Second, the proposed approach does not require any sophisticated and costly chemical conjugation protocols to generate functional BRET pair for PDT. Third, as showed in our studies, despite certain local toxic effects in organs due to furimazine injection, genetic encoding nature of synthesized protein complexes makes them safe and easily excretable from the organism, which is not the case for many chemically assembled structures. Finally, as probably the main advantage, the used principle of genetic encoding opens up novel appealing prospective for future improvements of PDT systems profiting from on genetic engineering approaches. In particular, the expression of used NanoLuc-miniSOG pair can be controlled on the genetic level by properly designed promoters (e.g., telomerase promoters), which are specific to some tumors. In addition, by fusing with a well-known protein localization motif (nuclear, membrane, mitochondrial) or a whole protein, the genetically encoded pair can be easily directed to any cell compartment (or even sub-compartment). Furthermore, the use of different targeting molecules in the composition of lentiviruses or other carriers renders possible an easy re-direction of a genetically encoded BRET-activated system on any tumor type, while the therapeutic action will address not only the primary tumor, but also its metastasis distributed in the organism. Thus, the employment of genetically encoded constructs provides novel opportunities to direct BRET-induced generation of active forms of oxygen to different cellular compartments or particular cell lines, which is hardly possible with chemically assembled BRET systems.

It should be noted that for the demonstration of targeted delivery of NanoLuc-miniSOG BRET pair to tumor cells, we used carriers based on lentiviruses. We believe that other organic or inorganic carries can be equally used to deliver genetically encoded PDT constructs to tumors and its metastasis. As one of attractive opportunities, we see the employment of promising nanomaterial systems, which can offer not only an additional tumor-targeting mechanism based on enhanced permeability and enhancement effect^35^, but also enable additional modalities for tumor imaging and therapy. As an example, the use of biodegradable laser-synthesized Si nanoparticles can offer additional optical imaging and photo- and radiofrequency radiation-induced therapy options^36^, while the use of titanium nitride nanoparticles can enrich the system by photothermal therapy effect^37^. To maximize the therapeutic outcome, such nanomaterial-based carrier systems can be further enhanced by the use of appropriate active targeting strategies, such as the one implying dual targeting by different protein-based vectors^38^. In general, use of genetically encoded BRET-activated constructs for PDT is a promising way to advance current state-of-the-art methods for non-invasive image-guided therapy of tumors and their metastasis.

We believe that the proposed genetically encoded BRET-activated PDT has a high potential for clinical translation. In this case, the use lentiviral particles seems most promising, as advanced molecular biology approaches make possible the generation of pseudotype LVs for any wished oncoreceptor. Moreover, taking into account heterogenic nature of tumor, one can use different pseudotype LVs recognizing different targets on the tumor cell surface.

The mechanism of cytotoxic action of PDT based on ROS generation is completely different from conventional chemotherapy and radiotherapy, that is why no cross-resistance between BRET-induced PDT and conventional therapy is known^39^. Owing to different mechanism of action, PDT can synergistically enhance chemotherapy. Moreover, some works reported that PDT leads to the sensitization of chemo- or radio-resistant cells making them sensitive to chemo- or radiotherapy^40,41^, including multidrug resistant cancers^42–46^. Therefore, BRET-PDT has a great potential for combination therapy.

In conclusion, we introduced the concept of genetically encoded BRET-activated PDT, which combines a triggering internal light source and a photosensitizer (PS) in a single-genetic construct, followed by a targeted delivery of the construct into cancer cells and BRET-mediated generation of ROS to initiate selective cancer cell kill. The concept is illustrated by using a home-designed NanoLuc-miniSOG platform, combining NanoLuc luciferase as the flashlight and phototoxic miniSOG as the PS, which is triggered by the injection of furimazine. We demonstrated the operation mechanism and assessed the efficiency of the concept by self-elimination of suicidal NanoLuc-miniSOG pair in BT-474 tumor cells implanted in mice, and then showed the possibility of targeted delivery of this pair, via lentiviral particles, into HER2-positive tumor, followed by its elimination. Presented results offer a new paradigm of how to generate photochemical effects in tissues without an external light source and in the absence of complex chemically assembled constructs. We believe that the proposed concept of genetically encoded BRET-activated PDT opens up the avenue for the development of a universal platform for synchronous delivery of light and PS in deep-seated tumors.

## Materials and methods

### Cell lines

HEK-293T cells (human embryonic kidney, ATCC CRL-3216), BT-474 cells (human ductal carcinoma, ATCC HTB-20) and BT-474 cells stably expressing NanoLuc-miniSOG in membrane localization or NanoLuc in the cytoplasm were cultured, as described previously^23^.

For the cultivation of miniSOG expressing cells, the medium was supplied with Riboflavin (Rf) (Pharmstandart-Ufa-Vita) to final concentration of 150 μM as a source of FMN cofactor for miniSOG.

### BRET measurement in vitro

The in vitro BRET measurement was carried out exactly as described previously^23^. Briefly, the emission spectra were recorded on living cells incubated in the medium without phenol red supplied with 75 μM of furimazine. Spectra were monitored and the data were processed, as described previously^23^. The BRET ratio calculation was performed as described by Eidne et al. ^47^.

### Fluorescence microscopy

To confirm NanoLuc-miniSOG expression in the membrane of BT-474, the experiment using a laser scanning microscope (Carl Zeiss LSM-710-NLO) was performed. Conditions of cell cultivation and imaging recording were the same, as previously described^23^.

### BRET-mediated in vitro phototoxicity

In vitro BRET-mediated phototoxicity was performed exactly as described previously^23^. Briefly, BT-474 cells with stable expression of NanoLuc-miniSOG in the plasma membrane were grown in the growth medium supplied with 150 μM Rf and treated with different luciferase-substrate concentrations (30, 43, or 75 μM furimazine). Cytotoxic effect was estimated using a standard MTT assay. The optical density was measured at 570 nm using Infinite 1000 Pro (Tecan). All experiments were repeated three times. Statistical analysis was performed using the Student’s t-test (unpaired).

### Estimation of ROS generation in vitro

Measurement of ROS production was carried on living BT-474 cells stably expressing NanoLuc-miniSOG in plasma membrane localization, as described previously^23^. The experiment was performed using a commercially available fluorescent dye carboxy-H_2_DCFDA (Invitrogen) according to manufacturer instructions.

### Tumor models and mice

Animals (six- to eight-week-old female BALB/c nu/nu athymic mice) were housed under the standard SPF conditions of the Animal Breeding Facility (branch of Shemyakin-Ovchinnikov Institute of Bioorganic Chemistry of the Russian Academy of Sciences). All experimental procedures were approved by the Animal Care and Use Committee of the Institute.

### Antitumor efficacy of NanoLuc-miniSOG-mediated PDT

BALB/c nu/nu mice were inoculated subcutaneously in the right flank with 10^7^ BT/NanoLuc-miniSOG-mem, BT/NanoLuc or 3*10^6^ BT-474 cells in 30% Matrigel (Corning) in 100 μL cultural media without serum and antibiotics per mouse. The tumor volume was calculated as described elsewhere^23^. When tumors reached ~100 mm^3^ (for experiment with mice bearing stably expressed NanoLuc-miniSOG or NanoLuc in xenograft tumors) or 50 mm^3^ (for lentiviral experiment), animals were assigned to the following treatment groups: control group treated with 0.1 mL PBS daily; groups with NanoLuc or NanoLuc-miniSOG expressing tumors treated intravenously (by injection in tail vein) with 7 μg of furimazine (Nano-Glo, Promega) and 10 mg/kg of Rf trice per day during 10 days, group with NanoLuc-miniSOG expressing tumors treated intravenously with 10 mg/kg of Rf trice per day during 10 days. The bodyweight of mice was measured once per 3 days. The part of the animals was euthanized the next day after the last injection and the remaining mice were euthanized on 23rd day after the first injection.

The tumor-growth inhibition coefficient (TGI) was calculated using standard formula^48^. Statistical analysis was performed using one-way ANOVA.

### Bioluminescent in vivo imaging

The experiments were carried out using an IVIS Spectrum CT system (Perkin Elmer, USA). If necessary, Rf was administered into the tail vein in the amount of 10 mg/kg per animal 1 h before administration of the substrate. Animals were anesthetized by isoflurane inhalation using the RAS-4 Rodent Anesthesia System (Perkin Elmer). For BRET study of NanoLuc-miniSOG system in vivo furimazine was i.v. administered at a dose of 20 μg per animal into the retroorbital sinus. Data acquisition was started 1 min after the substrate administration. To detect BRET in vivo, time dependence of signal intensity was monitored via a sequential acquisition of bioluminescent signal at an interval of about 2 min. All bioluminescence data have been collected in the mode “open filter” and normalized to the acquisition conditions and are displayed in radiance (photons/s/cm^2^/str). The values of the average intensity of the bioluminescent signal over the tumor area were used to plot the time dependence.

To detect direct fluorescence signal of miniSOG fluorescence in vivo, BT-474/Nano-Luc or NanoLuc-miniSOG-tumor-bearing mice were injected with Rf (10 mg/kg per animal in to the tail vein) if necessary. Then, furimazine was i.p. administered. Using IVIS Spectrum CT, fluorescence signals from 500 to 640 nm were collected using appropriate emission filters. In this experiment fluorescence was collected in “excitation block” mode, which means that miniSOG excitation was induced by oxidized form of NanoLuc substrate. To create fluorescence curves, the ROI on the first image was selected and plotted on all next images; all fluorescence data collected were normalized to the maximal fluorescence value (at 500 nm) and presented as average of radiance (photons/s/cm^2^/str).

For in vivo bioimaging of animals bearing HER2-positive BT-474 tumors transducted with LVs-NanoLuc-miniSOG or LVs-NanoLuc at 13th day after LVs injection, 6 μg furimazine per animal was i.p. administered and bioimaging was performed as described above.

### Isolation of a primary cell culture from a mouse tumor

A tumor of about 1 cm^3^ in size (approximately on the 25th day after inoculation) was removed under sterile conditions, dissected with a scalpel into 3–4 mm pieces, washed twice with 1 mL of the complete growth medium, resuspended in 1 mL of medium and incubated in the presence of 1 g/L collagenase type 4 and 1 g/L collagenase type 1 for 4 h at 37 °C. Then, a single-cell suspension was collected, precipitated by centrifugation, washed two times with a solution of PBS containing 1% BSA, and cultured under standard conditions in complete medium containing 10% fetal serum and antibiotics. After five passages, when the cell population became free from fibroblasts, cells were subjected to downstream assays.

### Plasmid constructs for lentiviral particles production

H-protein encoding plasmid pCG-Hc∆18-AA was a gift from Jakob Reiser (Addgene plasmid # 86559; http://n2t.net/addgene:86559; RRID: Addgene_86559), lentiviral packaging plasmid psPAX2 was a gift from Didier Trono (Addgene plasmid # 12260; http://n2t.net/addgene:12260; RRID: Addgene_12260). F-protein encoding plasmid pMD2-FΔ30 was constructed previously^49^. The DARPin-coding sequences have been PCR amplified as SmaI/NotI fragments using the primers dir (5′- TATCCCGGGATGGACCTGGGTAAGAAACTG -3′) and rev (5′- AATTGCGGCCGCTTGCAGGATTTCAGC -3′) from pET-DARP^48,50^. PCR product was ligated in-frame via SmaI/NotI into the plasmid pCG-Hc∆18-AA. NanoLuc-miniSOG-mem or NanoLuc coding sequences were amplified from plasmids pNanoLuc-miniSOG-mem^23^ or pNL1.1CMV (Promega, USA) using direct primer with BamHI site 5′-TACTGTGGATCCAGCCACCATGGTCTTCACAC and reveres primers with BamHI&SalI sites 5′-CAGGCGGATCCGTCGACTTCAGGAGAGCACACA or 5′-TAGCGGATCCGTCGACTCACGCCAGAATGCGTTCG, respectively, and cloned into pLCMV-Luc-puro^49^ instead of Luciferase gene.

### HER2-specific lentiviral particles production

Vector particles were generated, as described previously^31^. HEK-293T cells transfection was carried out using polyethyleneimine 25 kDA (PEI-25, Polysciences; USA) on Nunc 500 cm^2^ TripleFlasks and Nest 870 cm^2^ 5-layer flasks in OptiMEM medium (Invitrogen; USA). A mixture of the plasmids pLCMV-NanoLuc-miniSOG-mem-puro, pLCMV-NanoLuc-puro or tagRFP, psPAX2, pMD2-FΔ30, and pCG-HcΔ18 were used in a ratio of 8:8:7:1 in a total amount of 315 and 558 µg per 500 cm^2^ and 870 cm^2^ flask, respectively. Three hours later the medium with the transfection mixture was replaced with DMEM/F12 containing the serum replacement (Serum Replacement Solution and Lipid Mixture, PeproTech; USA), 2 mM alanyl-glutamine (PanEco; Russia), 20 mM HEPES and 4 mM caffeine. The following incubation was carried out up to 72 h for the production of viral particles. Viral particles were collected through 24, 48 and 72 h, and concentrated 500x by adding 1/3 of supernatant volume of PEG solution (40% PEG-8000 and 1.2 M NaCl), followed by overnight incubation on ice and subsequent centrifugation at 10,000 × *g* for 1 h at 4 °C. Viruses were injected into the BT-474 tumors of mice immediately after concentrating in amount of ~5*10^6^ IFU as measured on BT-474 cells.

Vector particles pseudotyped with the VSV-G protein were produced by co-transfection of 4.55 μg of the plasmid pMD2.G (a gift from Didier Trono, Addgene plasmid #12259; http://n2t.net/addgene:12259; RRID:Addgene_12259), along with 8.45 μg of psPAX2 and 13.00 μg of transfer plasmid.

### DARPin conjugation to FITC

500 nM of DARPin_9-29 in 20 mM Na-phosphat buffer, pH 8.0 was incubated with fivefold molar excess of FITC (Sigma) for 2 h at room temperature. To remove the unbound dye, the reaction mixture was passed through a desalting column (NAP-5, GE Healthcare).

### Flow cytometry

Forty-eight hours after transduction of BT-474 or MDA-MB-231 cells with VSV-tagRFP or DARPin_9-29-tagRFP LVs, the percentage of tagRFP-positive cells in population was measured by Novocyte 3000 VYB flow cytometer (ACEA Biosciences, USA) in YL1 channel (excitation laser 561 nm, emission filter 586/20 nm).

To assess the HER2 expression levels on BT-474 or MDA-MB-231 cells, they were stained with DARPin_9-29 conjugated with FITC as follows. Cells (10^6^ cells per mL) transduced with VSV-tagRFP or DARPin_9-29-tagRFP LVs, were incubated with DARPin_9-29-FITC (100 nM) in PBS supplemented with 1% BSA for 15 min at 37 °C. Standard washing procedure was carrried out befor measurement. TagRFP^+^/FITC^+^ double positive cells were determined by Novocyte 3000 VYB flow cytometer. FITC fluorescence was measured using 488 nm exitation laser and 530/30 nm emission filter.

### Histological analysis

For histological examination, 24 h after the last treatment tumors and organs (spleen, liver, heart, lungs, kidneys and lymph nodes) were surgically removed and fixed in 4% neutral-buffered formalin, dehydrated and embedded in paraffin. Three-micrometer sections made on rotary microtome Leica RM2255 were stained with hematoxylin and eosin, covered with Acrytol and examined with light microscopy. The study was carried out at the optical level using a DMI6000B microscope (Leica Microsystems CMS, Germany) at magnification ×100, ×200, ×400.

## Data Availability

All data needed to evaluate the conclusions in the paper are present in the paper. Additional data related to this paper may be requested from the authors.
